# The Effects of Selenium Supplementation in the Treatment of Autoimmune Thyroiditis: An Overview of Systematic Reviews

**DOI:** 10.3390/nu15143194

**Published:** 2023-07-19

**Authors:** Yong-Sheng Wang, Shan-Shan Liang, Jun-Jie Ren, Zi-Yi Wang, Xin-Xin Deng, Wen-Di Liu, Yi-Long Yan, Gui-Hang Song, Xiu-Xia Li

**Affiliations:** 1 Evidence-Based Social Science Research Center/Health Technology Assessment Center, School of Public Health, Lanzhou University, Lanzhou 730000, China; wangysh18@lzu.edu.cn (Y.-S.W.); liangshsh18@lzu.edu.cn (S.-S.L.); renjj18@lzu.edu.cn (J.-J.R.); wangzy2018@lzu.edu.cn (Z.-Y.W.); 220220912290@lzu.edu.cn (X.-X.D.); 220220912620@lzu.edu.cn (W.-D.L.); yanyl2018@lzu.edu.cn (Y.-L.Y.); 2 Key Laboratory of Evidence Based Medicine and Knowledge Translation of Gansu Province, Lanzhou University, Lanzhou 730000, China; 3 Gansu Healthcare Security Administration, Lanzhou 730000, China; 220220913531@lzu.edu.cn

**Keywords:** selenium, thyroid, autoimmune thyroiditis, overview, meta-analysis

## Abstract

Objective: The available evidence on selenium supplementation in the treatment of autoimmune thyroiditis (AIT) was inconclusive. This research serves to assess the effects of selenium supplementation in the treatment of AIT. Methods: Online databases including PubMed, Web of Science, Embase, and the Cochrane Library were searched from inception to 10 June 2022. The AMSTAR-2 tool was used to assess the methodological quality of included studies. The information on the randomized controlled trials of the included studies was extracted and synthesized. The GRADE system was used to assess the certainty of evidence. Results: A total of 6 systematic reviews with 75 RCTs were included. Only one study was rated as high quality. The meta-analysis showed that in the levothyroxine (LT4)-treated population, thyroid peroxidase antibody (TPO-Ab) levels decreased significantly in the selenium group at 3 months (SMD = −0.53, 95% CI: [−0.89, −0.17], *p* < 0.05, very low certainty) and 6 months (SMD = −1.95, 95% CI: [−3.17, −0.74], *p* < 0.05, very low certainty) and that thyroglobulin antibody (Tg-Ab) levels were not decreased. In the non-LT4-treated population, TPO-Ab levels decreased significantly in the selenium group at 3 and 6 months and did not decrease at 12 months. Tg-Ab levels decreased significantly in the selenium group at 3 and 6 months and did not decrease at 12 months. The adverse effects reported in the selenium group were not significantly different from those in the control group, and the certainty of evidence was low. Conclusion: Although selenium supplementation might reduce TPO-Ab levels at 3 and 6 months and Tg-Ab levels at 3 and 6 months in the non-LT4-treated population, this was based on a low certainty of evidence.

## 1. Introduction

Autoimmune thyroiditis (AIT) is a chronic autoimmune disease in which human thyroid tissue serves as an antigen. AIT involves the production of autoantibodies such as thyroid peroxidase antibody (TPO-Ab) and thyroglobulin antibody (Tg-Ab), which may trigger cellular and antibody-mediated immune processes that lead to the destruction of thyroid cells [1]. Clinical manifestations include goiter, pharyngeal discomfort, neck compression, and dysphagia [2]. AIT includes Hashimoto’s thyroiditis (HT), Graves’ disease (GD) and other diseases. Lymphocyte infiltration in HT can gradually destroy follicular cells and lead to hypothyroidism [3]. AIT affects about 5% of the general population and its incidence rate in women is about 4–10 times that in men; the incidence rate increases with age [3,4]. Currently, it is unclear whether the incidence rate of AIT is increasing. However, the prevalence of hypothyroidism may be decreasing due to the widespread use of levothyroxine (LT4) [5,6]. Various studies have proved that there is a relationship between AIT and thyroid cancer, so it is necessary to actively intervene in AIT [7]. For AIT patients with hypothyroidism, the current standard therapy is LT4 replacement therapy to maintain normal thyroid function [7]. The relevant guidelines suggest that patients should receive treatment when thyroid-stimulating hormone (TSH) levels are above 10 mIU/L [8,9].

Selenium is an essential micronutrient for the human body. Very low selenium concentration is associated with numerous diseases, such as endemic osteoarthropathy (Kashin–Beck disease) and dilated cardiomyopathy (Keshan disease) [10]. Selenium plays a critical role in thyroid function; the thyroid is one of the organs with the highest levels of selenium in the body [11]. Some studies have shown that antioxidants may have therapeutic effects in preventing AIT and selenium is significant in antioxidation [1]. Selenium is essential in the molecular structure of thyroid enzymes, including glutathione peroxidase, which defends the thyroid against the oxidative damage caused by hydrogen peroxide enzyme synthesis by thyroid hormones [12]. All these factors indicate that selenium has broad application prospects in the treatment and prognosis of AIT. Selenium, as a complementary therapy, may reduce antibody levels and decrease doses of LT4 use [13]. The relevant guideline indicated that for mild Graves’ orbitopathy, selenium may improve ocular manifestations and quality of life, potentially preventing disease progression [14].

Previous relevant studies have explored the relationship between selenium supplementation and the treatment of AIT but their findings were inconclusive. In a Cochrane review from 2013 that was based on four randomized controlled trials (RCTs), it was concluded that the evidence supporting the effects of selenium supplementation in Hashimoto’s thyroiditis patients was incomplete [13]. Some systematic reviews (SRs) showed that selenium supplementation was effective in treating AIT. Zuo 2021, based on 17 RCTs, considered that selenium could significantly reduce the levels of TPO-Ab in AIT patients and selenium-containing drugs could effectively treat AIT [2]. Fan 2014, based on nine RCTs, pointed out that selenium supplementation could significantly reduce the TPO-Ab levels at 6 and 12 months and that the Tg-Ab levels could decrease at 12 months. Patients had a higher chance of improving their mood with no obvious adverse effects. Selenium supplementation proved to be an effective complementary therapy for AIT [15]. An investigation showed that in actual clinical practice in some regions, more than 80% of doctors at least sometimes prescribed selenium supplementation for AIT patients, which was beyond the evidence support of evidence-based medicine [16].

We conducted an overview of the systematic reviews of selenium supplementation in treating AIT. At the same time, the RCTs included in SRs were extracted for data synthesis. Our purpose was to further summarize whether selenium supplementation was an effective treatment method for AIT and thus provide assistance for clinical practice.

## 2. Materials and Methods

### 2.1. Registration of Overview of Systematic Reviews

The study was registered at PROSPERO on 29 July 2022 (registration number: CRD42015025247).

### 2.2. Inclusion and Exclusion Criteria

We included SRs and meta-analyses based on RCTs. Studies meeting the following criteria were included: (1) For adults (≥18 years old) diagnosed with autoimmune thyroiditis, the diagnostic criteria may be varied in different regions but is generally established by elevated TSH and normal free thyroxine (FT4), TPO-Ab positivity, or decreased echogenicity of the thyroid parenchyma observed on ultrasonography. (2) Intervention measures included selenium supplementation, which could be combined with standard therapy. Control measures could be blank control, placebo control, or standard therapy without selenium supplementation. Selenium supplementation included selenomethionine, sodium selenite, selenium yeast, and other forms, with unlimited dosage. (3) The main outcomes included TPO-Ab levels and/or Tg-Ab levels. Exclusion criteria included the following: (1) duplicate reports; (2) studies with insufficient data; (3) thyroid disease during pregnancy; (4) thyroid-associated ophthalmopathy; (5) studies not in English or Chinese.

### 2.3. Search Methods

Online databases including PubMed, Web of Science, Embase, and the Cochrane Library were searched from inception to 10 June 2022, and the retrieval was performed again before the final data analysis. MeSH words and keywords were combined in the search strategy. The main search strategy was as followed (take PubMed search strategy as an example): (“thyroiditis, autoimmune” [MeSH] OR “graves disease” [MeSH] OR “autoimmune thyroiditis” OR “AIT” OR “ATD” OR “hashimoto thyroiditis” OR “HT” OR “hashimoto disease” OR “painless thyroiditis” OR “graves disease” OR “GD” OR “lymphocytic thyroiditis” OR “hyperthyroidism”) AND (“selenium” [MeSH] OR “selenium compounds” [MeSH] OR “organoselenium compounds” [MeSH] OR “selen*” OR “Se” OR “ebselen”) AND (“systematic reviews as Topic” [MeSH] OR “Meta-Analysis as Topic” [MeSH] OR “meta analys*” OR “systematic revie*” OR “metaanalys*”).

### 2.4. Study Selection and Data Extraction

Two reviewers independently evaluated whether the articles met the inclusion criteria. Any disagreements were discussed with a third reviewer and resolved by consensus. This process was documented in the PRISMA flowchart [17].

For the included studies, the two reviewers used the pre-designed extraction table to independently extract the data from each study and crosscheck them. The data extraction content included information such as the first author, year of publication, sample size, research object, intervention measures, main report outcomes, and main results. At the same time, our study extracted the data from RCTs included in the SRs, including the first author, year of publication, sample size, intervention measures, TPO-Ab and Tg-Ab levels of baseline and treatment endpoint, adverse effects, age, gender, etc.

### 2.5. Assessment of Methodological Quality

We used the measurement tool to assess systematic review 2 (AMSTAR-2) [18] to evaluate the quality of the methodology of the SRs. AMSTAR-2 included 16 evaluation items, of which, seven were critical items, where “Y” represented conformity, “N” represented non-conformity, “PY” represented partial conformity, and “NP” represented not applicable because no meta-analysis was conducted. The quality assessment was completed by an online tool (https://amstar.ca/Amstar_Checklist.php, accessed on 2 September 2022), and the overall quality of the study was automatically generated after the assessment was completed. Each study was evaluated as high, moderate, low, or critically low quality. Two reviewers independently evaluated the studies and any discrepancies were discussed with a third reviewer and resolved by consensus.

### 2.6. Certainty Assessment

We used the grades of recommendation, assessment, development, and evaluation (GRADE) system to assess the certainty of evidence and constructed a summary of findings table. The evaluation content included five factors: risk of bias, inconsistency, indirectness, imprecision, and publication bias [19]. Among them, the Cochrane Collaboration risk of bias tool (CCRBT) was used for risk of bias assessment.

### 2.7. Data Synthesis and Analysis

We performed a descriptive analysis of the included SRs. The document management software EndNote 20 was used to import, screen, and manage documents and remove duplicates, and Excel 2021 was used to design data extraction tables and implement data statistics.

For the RCTs included in the SRs, STATA 17 was used for data synthesis. The standardized mean difference (SMD) was used for data synthesis for continuous variables because the trials used various measurement scales to measure the same outcomes and relative risk (RR) was used for data synthesis for binary variables. Heterogeneity was assessed using the Q-test and *I*^2^ statistics. *p* < 0.05 and *I*^2^ > 50% indicated significant heterogeneity and used a random-effect model. Otherwise, a fixed-effect model was selected. Subgroup analysis was conducted based on the type of intervention measures and trial duration. Sensitivity analysis was conducted by “Leave-one-out” to assess the impact of each study on the effect size of meta-analysis [20], so as to test the robustness of the meta-analysis. Egger’s test was used to evaluate publication bias.

## 3. Results

### 3.1. Study Selection

**Figure 1:**
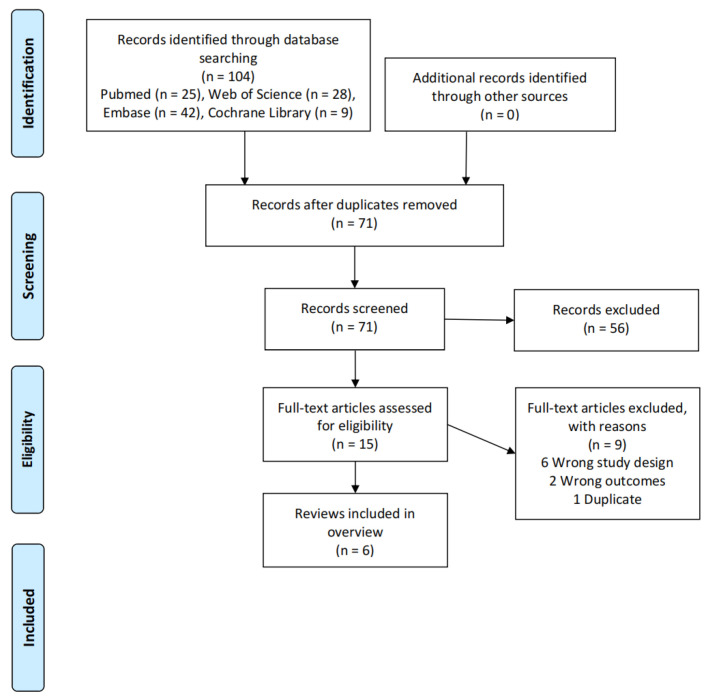
Literature screening process.

A total of 104 relevant records were identified, and 71 records were obtained after removing duplicates. A total of 56 records were excluded by reading the title and abstract. A total of 15 records were further screened by reading the full text, of which, six records were ineligible for study design, two records were ineligible for outcomes, and one record was repeated. Finally, six studies were included in the overview of review [2,13,15,21,22,23]. The flow diagram of the literature screening is displayed in Figure 1.

### 3.2. Study Characteristics

The included studies were published between 2010 and 2021, including RCTs published until November 2020. Seven outcomes were reported, including TPO-Ab, Tg-Ab, TSH, free triiodothyronine (FT3), FT4, mood/wellbeing, immunomodulatory effects, and adverse effects. Five of the six studies conducted meta-analysis. The CCRBT was used in four studies, the Jadad scale was used in one study, and the quality assessment tool was not used in one article. The essential characteristics of the SRs are shown in Table 1.

### 3.3. Assessment of Methodological Quality

**Figure 2:**
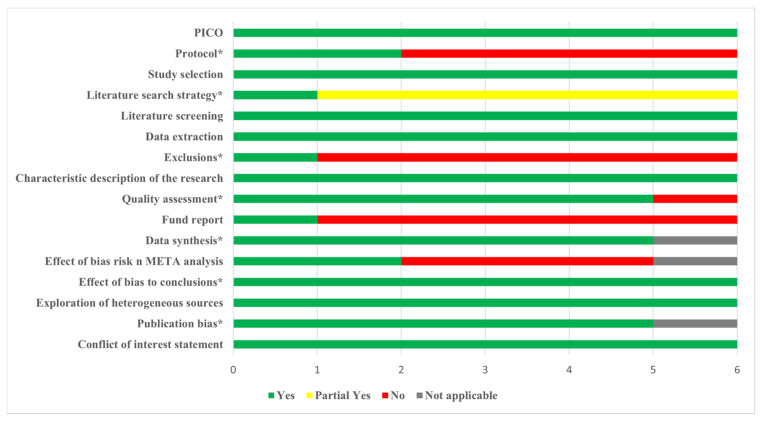
Compliance of the AMSTAR-2 items (* critical item).

The AMSTAR-2 results showed one study of high quality, one study of low quality, and four studies of critically low quality. The compliance status of each item is shown in Figure 2. The absence of a protocol for the study specified before the start of the review and the lack of a list of excluded documents and the reasons for their exclusion were the main critical flaws. The non-critical weakness was that the source of funding for the studies included in the review were not reported. The AMSTAR-2 results for each SR are shown in Table S1.

### 3.4. Characteristics and Risk of Bias of RCTs

A total of 75 RCTs were included in six SRs. After excluding duplicate and ineligible trials, 23 RCTs were included for data synthesis [24,25,26,27,28,29,30,31,32,33,34,35,36,37,38,39,40,41,42,43,44,45,46]. The detailed characteristics are documented in Table S2. Three trials had two intervention groups and two control groups that were divided into trials A and B based on whether they received LT4 treatment. One trial described that the outcome data were divided into groups with restored/non-restored thyroid function, which were combined into one [20]. A total of 23 RCTs including a total of 2292 patients, 89.7% of whom were women.

**Figure 3:**
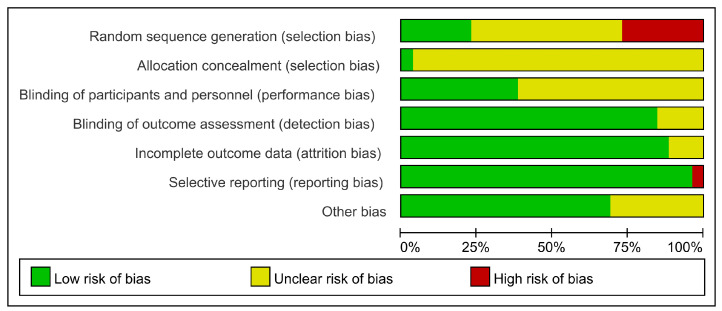
The risk of bias for RCTs.

In random sequence generation, 19 trials reported the utilization of randomization, in which 13 trials did not report the specific randomized methods and were therefore assessed as unclear risk of bias; four trials did not report the use of randomization and were assessed as high risk of bias. Only one trial clarified the allocation concealment and was assessed as low risk of bias. A total of 12 trials did not specify the blinding of participants and personnel, so they were assessed as unclear risk of bias. Because most outcomes were objective antibody levels, which were unlikely to cause bias, 19 trials were assessed as low risk of bias in the blinding of outcome assessment. Three trials were assessed as unclear risk of bias in incomplete outcome data due to insufficient information. In selective reporting, one trial was assessed as high risk of bias due to the differences between the designs and results. Eight trials could not assess whether there was any other bias affecting the results because there was not enough information, so they were assessed as unclear risk of bias (Figure 3). Figure S1 shows the bias risk for each RCT.

### 3.5. Meta-Analysis

#### 3.5.1. Change in TPO-Ab Levels

**Figure 4:**
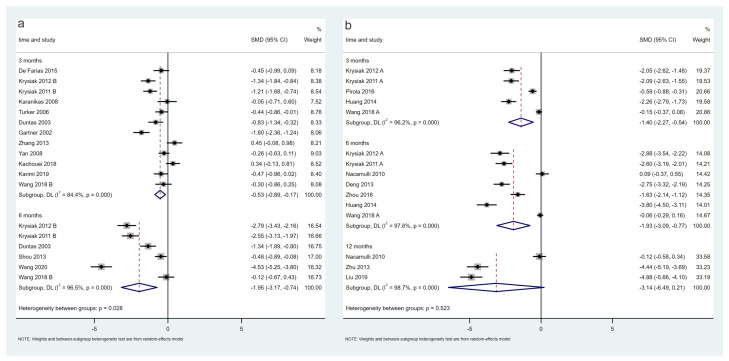
Difference of TPO-Ab levels between the selenium group and control group in the LT4 (**a**) and non-LT4 treatment groups (**b**). The black block represents the effect sizes of individual studies, red dashed line represents combined effect sizes, blue diamond block represents the 95%CI of combined effect sizes [24,26,27,28,29,31,32,33,34,35,36,37,38,39,40,41,42,43,44,45,46].

A total of 14 trials, including 1041 patients, evaluated changes in TPO-Ab levels in the LT4-treated population. The meta-analysis results showed that TPO-Ab levels in the selenium group decreased significantly at 3 months (12 trials, SMD = −0.53, 95% CI: [−0.89, −0.17], *p* < 0.05) and 6 months (six trials, SMD = −1.95, 95% CI: [−3.17, −0.74], *p* < 0.05) (Figure 4a). The certainty in the evidence was very low at 3 and 6 months (Table 2).

Ten trials, including 1198 patients, evaluated the change in TPO-Ab levels in the non-LT4-treated population. The meta-analysis results showed that TPO-Ab levels in the selenium group decreased significantly at 3 months (five trials, SMD = −1.40, 95% CI: [−2.27, −0.54], *p* < 0.05) and 6 months (six trials, SMD = −1.93, 95% CI: [−3.09, −0.77], *p* < 0.05) and not significantly at 12 months (*p* > 0.05) (Figure 4b). The certainty in the evidence was low at 3 and 6 months and very low at 12 months (Table 2). The sensitivity analysis showed that the association between selenium supplementation and TPO-Ab levels at 12 months in the non-LT4-treated group was fragile. When Nacamulli 2010 [28] was excluded, TPO-Ab levels decreased significantly and *I*^2^ reduced to 0%. This was because the outcome data for this study were presented as the median and 95% CI, and the calculated mean and SD might not be reliable.

#### 3.5.2. Change in Tg-Ab Levels

**Figure 5:**
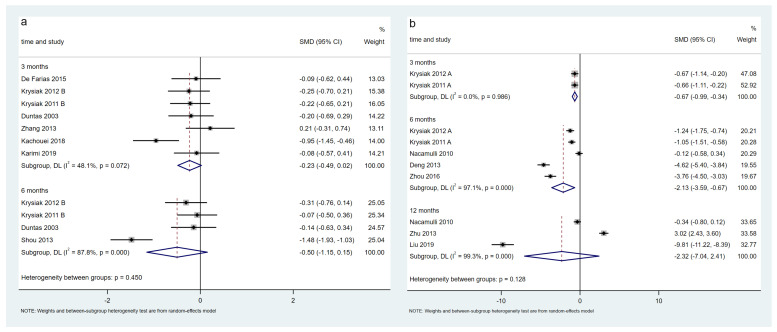
Difference in Tg-Ab levels between the selenium group and control group in LT4 (**a**) and non-LT4 treatment groups (**b**). The black block represents the effect sizes of individual studies, red dashed line represents combined effect sizes, blue diamond block represents the 95%CI of combined effect sizes [24,26,27,28,32,34,35,36,38,39,40,44,45].

Eight trials, including 577 patients, evaluated changes in Tg-Ab levels in the LT4-treated population. The meta-analysis results showed that Tg-Ab levels of the selenium group did not decrease significantly at 3 and 6 months (*p* > 0.05) (Figure 5a). The certainty in the evidence was low at 3 months and very low at 6 months (Table 2). Two trials randomized and stratified the patients according to the baseline TPO-Ab levels, so the Tg-Ab levels at baseline were not comparable and were excluded from the meta-analysis [31,33]. The association between the selenium supplementation and Tg-Ab levels at 3 months in the LT4-treated population was also fragile. When Zhang 2013 [36] was excluded, the TG-Ab levels significantly reduced and *I*^2^ reduced to 39%, which might be due to the lack of specificity of Tg-Ab in HT.

Seven trials, including 603 patients, evaluated changes in Tg-Ab levels in the non-LT4-treated population. The meta-analysis results showed that Tg-Ab levels in selenium group decreased significantly at 3 months (two trials, SMD = −0.67, 95% CI: [−0.99, −0.34], *p* < 0.05) and 6 months (five trials, SMD = −2.13, 95% CI: [−3.59, −0.67], *p* < 0.05) and not significantly at 12 months (*p* > 0.05) (Figure 5b). The certainty in the evidence was moderate at 3 months, low at 6 months, and very low at 12 months (Table 2).

#### 3.5.3. Adverse Effects

**Figure 6:**
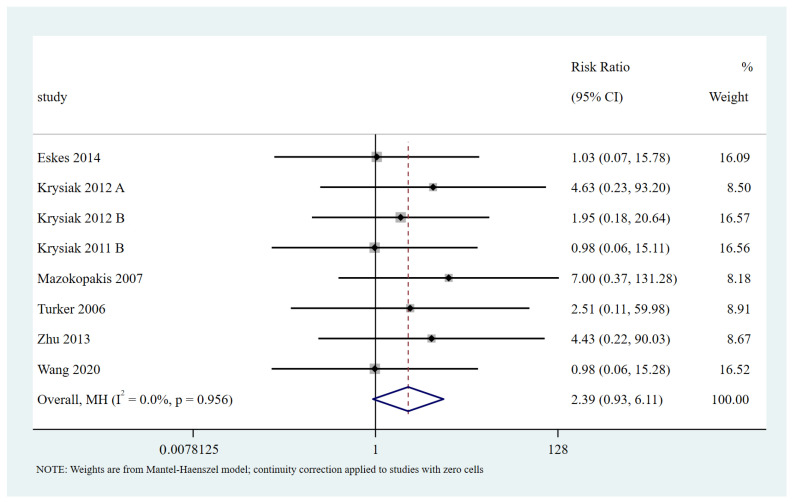
Difference of adverse effects between the selenium group and control group. The black block represents the effect sizes of individual studies, red dashed line represents combined effect sizes, blue diamond block represents the 95%CI of combined effect sizes [25,26,27,30,31,35,41].

Eight trials, including 669 patients, evaluated the adverse effects. The most common adverse effect was gastric discomfort (10 in the selenium group and one in the control group); other adverse effects included hair loss (one in the selenium group and one in the control group), headache (one in the selenium group), skin rash (one in the selenium group), and hyperthyroidism (two in the control group). No serious adverse effects were observed. The was no statistically significant difference in the risk of adverse effects between the selenium and control groups (RR = 2.39, 95% CI: [0.93 to 6.11]; *p* > 0.05) (Figure 6). The certainty in the evidence was low (Table 2). Three RCTs reported glucose or HbA1c [26,27,34], and the results showed that there were no significant differences in glucose or HbA1c concentrations between the selenium and control groups.

#### 3.5.4. Publication Bias

Egger’s test indicated no publication bias in the TPO-Ab levels at 3 months in the LT4-treated population (Egger’s test, *p* = 0.755). The publication bias of other results could not be evaluated because the meta-analysis of the other results did not include more than 10 trials.

### 3.6. Summary of Findings

The GRADE system was used to grade the certainty of evidence for each outcome and a summary of findings table was constructed (Table 2). The outcomes of TPO-Ab and Tg-Ab levels were both downgraded due to their indirectness, as they involved surrogate markers for clinical efficacy or disease progression [47].

## 4. Discussion

Our study included SRs before 10 June 2022 and the RCTs included in SRs were published until November 2020. Of the six included studies, two were published in the last 5 years, which showed that AIT was receiving increasing attention and there was more evidence for selenium supplementation to treat AIT. However, different evidence led to different conclusions. Therefore, this study conducted an overview of the reviews on selenium supplementation for treating AIT to evaluate the quality of the existing studies and further summarize the current evidence, providing a reference for clinical practice.

According to the findings of AMSTAR-2, the methodological quality of the SRs included in this study was not good, with only one study of high quality, one study of low quality, and four studies of critically low quality. As for critical items, in Item 2, only two studies specified the research protocol before the SRs began. The registration of the SR before the research can not only reduce the risk of bias and improve the report quality but also save research resources [48]. Other SR authors could determine whether the research was repeated by searching the registration platform. In Item 4, five studies partially met the requirements of the comprehensive literature search strategy but did not fully meet the requirements due to the lack of supplemented retrieval by reviewing the reference list from the studies found, the lack of searching relevant gray literature, the lack of a complete search strategy, and other reasons. Other researchers might not be able to reproduce the search results, reducing the reliability of the results. In Item 7, five studies failed to provide a complete list of excluded studies, which might cause omission when screening the literature. For non-critical items, five studies in Item 10 did not provide the funding source for the included studies, which might ignore some risks of bias. The above were the main items that affected the methodological quality included in this study; future research should focus on the quality of methodology [49].

In addition, we extracted and synthesized the data from RCTs included in SRs. The CCRBT showed that the current randomized controlled trials for selenium therapy in AIT could still have improved study design. A total of 19 trials reported the use of randomization, but 13 did not specify the specific randomized method. Only one trial clarified the allocation concealment. This also led to a downgrade in the risk of bias in the GRADE system. In the future, RCTs can utilize CCRBT to improve the quality of research.

According to previous experience, receiving LT4 treatment might affect the outcome, so this study divided the patients into two groups according to whether they received LT4 treatment for data synthesis [21]. The results showed that in the LT4-treated population, the TPO-Ab levels in the selenium group decreased at 3 and 6 months but the Tg-Ab levels did not decrease. In the non-LT4-treated population, the TPO-Ab levels in the selenium group decreased at 3 and 6 months and did not decrease at 12 months. The Tg-Ab levels decreased at 3 and 6 months and did not decrease at 12 months. The decreases in TPO-Ab levels in the LT4-treated population and the non-LT4-treated population were consistent with the meta-analysis results of Qiu 2020 et al. [23]. The insignificant decrease in Tg-Ab levels might be due to the lack of specificity in HT [36]. It is worth noting that the association between TPO-Ab levels at 12 months in the non-LT4-treated population and Tg-Ab levels at 3 months in the LT4-treated population and selenium supplementation was not reliable.

The selenium treatment and control groups showed no significant differences in adverse effects, and no serious adverse effects were observed. This showed that selenium supplementation was a safe and effective treatment for AIT. This result was consistent with the results of Fan 2014 et al. [15] but inconsistent with the results of Qiu 2020 et al. [23]. These two studies were based on two and five trials, respectively, whereas our study was based on more RCTs, so the results might be more reliable. Most of the selenium supplementation used in the trials was 200 μg/day, and 200 μg of selenomethionine was equivalent to 80 μg of selenium [24]. According to relevant studies, the recommended intake dose of selenium is 55 μg/day and the tolerable upper limit is 400 μg/day [50]. The current selenium dose used in the trials was reasonable. Some studies have shown that high levels of selenium intake are associated with the development of diabetes [51]. In the studies we included, no such trend was observed. In addition, the selenium supplementation selected in the current RCTs was mainly in the form of selenium salts (sodium selenite), amino acids (selenomethionine), and selenium yeast. There was a new generation of selenium supplements, including zerovalent selenium nanoparticles and selenized polysaccharides, that had the advantages of low toxicity, high bioavailability, and controlled release [52]. They could be considered for use in future research.

The certainty of evidence in the GRADE system showed that the outcomes for TPO-Ab and Tg-Ab levels were both graded as low or very low; only the difference in Tg-Ab levels at 3 months in the non-LT4-treated population was of moderate certainty. Adverse effects were graded as low certainty. This indicated that there might be some differences between the current results and the real situation and that the current results need to be treated with caution. Further research needs to be carried out in the future.

This study had some limitations as well. Limited by language barriers, we were only able to search English databases. Due to the characteristics of the study design, even recently published SRs were unlikely to be included the latest literature, so our study omitted this literature after November 2020. In addition, the meta-analysis of the RCTs showed significant heterogeneity. Firstly, the study included various types of AIT disease rather than focusing on one specific type, resulting in some clinical heterogeneity. Secondly, different selenium preparations and doses posed challenges in comparing different studies. Thirdly, the study estimated some results using the median and IQR and the median and 95% CI, which may not be reliable and could impact the results.

## 5. Conclusions

Although selenium supplementation could reduce the TPO-Ab levels at 3 and 6 months and the Tg-Ab levels at 3 and 6 months in the non-LT4-treated population, the routine use of selenium supplementation in patients with AIT is not recommended due to the low certainty of evidence. In current clinical practice, selenium supplementation beyond the support of evidence-based medical evidence should be corrected. Since low selenium status is closely related to many diseases, it is feasible to supplement selenium only in patients with selenium deficiency. In the future, it is expected that RCTs with rigorous design, long-term follow-up, and using the new generation of selenium supplementation will offer high-quality evidence to inform clinical decision making.

## Figures and Tables

**Table 1:** The essential characteristics of the included studies.

Study	Documents (Sample Size)	Research Object	Intervention Measures		Main Report Outcome *	Quality Assessment Tool	Main Results
			Intervention Group	Control Group			
Wichman 2016 [21]	16(1494)	AIT	Selenium alone or in combination with LT4	Blank control/placebo alone or in combination with LT4	①②⑦⑧	CCRBT	In the LT4-treated population, TPO-Ab and Tg-Ab levels in the selenium group decreased after 3, 6, 12 months, and 12 months, respectively; in the non-LT4-treated population, TPO-Ab and Tg-Ab levels in the selenium group decreased after 3 months. It still needs to be demonstrated whether these effects were associated with clinical measures.
van Zuuren 2013 [13]	4(463)	HT	Selenium alone or in combination with LT4	Blank control/placebo alone or in combination with LT4	①⑥⑧	CCRBT	The evidence regarding the efficacy of selenium supplementation in patients with HT was insufficient.
Fan 2014 [15]	9(797)	AIT	Selenium alone or in combination with LT4 or methimazole	Blank control/placebo alone or combined with LT4 or methimazole	①②⑥⑧	Jadad scale	Selenium supplementation was related to the significant decrease in TPO-Ab levels at 6 and 12 months; at the same time, the Tg-Ab levels could decrease at 12 months. After selenium supplementation, patients had an increased probability to improve their mood without obvious adverse events.
Toulis 2010 [22]	6(339)	HT	Selenium alone or in combination with LT4	Placebo alone or in combination with LT4	①⑥⑦⑧	None	Selenium supplementation was linked to a significant reduction in TPO-Ab levels at 3 months, as well as an improvement in mood and/or general wellbeing.
Zuo 2021 [2]	17(1911)	AIT	Selenium alone or in combination with LT4 or methimazole	Blank control/placebo alone or combined with LT4 or methimazole	①②③④⑤	CCRBT	Selenium-containing medications demonstrated effectiveness in treating AIT patients and significantly reduced the levels of TPO-Ab in AIT patients. TSH and TG-Ab had no notable difference between the selenium and control group.
Qiu 2020 [23]	23(2394)	AIT	Selenium alone or in combination with LT4	Blank control/placebo alone or in combination with LT4	①②③⑦⑧	CCRBT	In the LT4-treated population, TPO-Ab and Tg-Ab levels in the selenium group decreased after 3, 6, 12 months, and 12 months, respectively; in the non-LT4-treated population, TPO-Ab levels in the selenium group decreased after 3 and 6 months and Tg-Ab levels decreased after 3 months. Based on the current evidence, there was insufficient justification for the new use of selenium supplementation in the treatment of AIT.

* Main report outcome ① TPO-Ab; ② Tg-Ab; ③ TSH; ④ FT3; ⑤ FT4; ⑥ mood/wellbeing; ⑦ Immunomodulatory effects; ⑧ Adverse effects. Abbreviations: AIT, Autoimmune thyroiditis; HT, Hashimoto’s thyroiditis; LT4, levothyroxine; CCRBT, Cochrane Collaboration risk of bias tool; TPO-Ab, thyroid peroxidase antibody; TG-Ab, thyroglobulin antibody.

**Table 2:** Summary of findings.

Outcomes	Anticipated Absolute Effects * (95% CI)		Relative Effect (95% CI)	No. of Participants	Certainty of the Evidence (GRADE)
	Risk with Placebo	Risk with Selenium			
TPO-Ab (LT4-treated population, 3 months)	-	SMD 0.53 lower (0.89 lower to 0.17 lower)	-	840 (12 trials)	⨁◯◯◯ Very low ^a,b,c^
TPO-Ab (LT4-treated population, 6 months)	-	SMD 1.95 lower (3.17 lower to 0.74 lower)	-	476 (6 trials)	⨁◯◯◯ Very low ^a,b,c^
TPO-Ab (Non-LT4-treated population, 3 months)	-	SMD 1.40 lower (2.27 lower to 0.54 lower)	-	750 (5 trials)	⨁⨁◯◯ Low ^b,c^
TPO-Ab (Non-LT4-treated population, 6 months)	-	SMD 1.93 lower (3.09 lower to 0.77 lower)	-	808 (7 trials)	⨁⨁◯◯ Low ^b,c^
TPO-Ab (Non-LT4-treated population, 12 months)	-	SMD 3.14 lower (6.49 lower to 0.21 higher)	-	274 (3 trials)	⨁◯◯◯ Very low ^b,c,d^
TG-Ab (LT4-treated population, 3 months)	-	SMD 0.23 lower (0.49 lower to 0.02 higher)	-	481 (7 trials	⨁⨁◯◯ Low ^c,d^
TG-Ab (LT4-treated population, 6 months)	-	SMD 0.50 lower (1.15 lower to 0.15 higher)	-	320 (4 trials)	⨁◯◯◯ Very low ^a,b,c,d^
TG-Ab (Non-LT4-treated population, 3 months)	-	SMD 0.67 lower (0.99 lower to 0.34 lower)	-	155 (2 trials)	⨁⨁⨁◯ Moderate ^c^
TG-Ab (Non-LT4-treated population, 6 months)	-	SMD 2.13 lower (3.59 lower to 0.67 lower)	-	405 (5 trials)	⨁⨁◯◯ Low ^b,c^
TG-Ab (Non-LT4-treated population, 12 months)	-	SMD 2.32 lower (7.04 lower to 2.41 higher)	-	274 (3 trials)	⨁◯◯◯ Very low ^b,c,d^
Adverse effects	12 per 1000	38 per 1000 (12 to 79)	RR 2.93 higher (0.93 higher to 6.11 higher)	669 (8 trials)	⨁⨁◯◯ Low ^a,d^

* The risk in the intervention group (and its 95% confidence interval) is based on the assumed risk in the comparison group and the relative effect of the intervention (and its 95% CI). Abbreviations: SMD, standardized mean difference; RR, risk ratio. GRADE Working Group grades of evidence: High certainty (⨁⨁⨁⨁): We are very confident that the true effect lies close to that of the estimate of the effect. Moderate certainty (⨁⨁⨁◯): We are moderately confident in the effect estimate: The true effect is likely to be close to the estimate of the effect, but there is a possibility that it is substantially different. Low certainty (⨁⨁◯◯): Our confidence in the effect estimate is limited: The true effect may be substantially different from the estimate of the effect. Very low certainty (⨁◯◯◯): We have very little confidence in the effect estimate: The true effect is likely to be substantially different from the estimate of effect. ^a^ Downgraded one level because of risk of bias. ^b^ Downgraded one level because of inconsistency. ^c^ Downgraded one level because of indirectness. ^d^ Downgraded one level because of imprecision, including wide confidence intervals, lack of participants, and lack of events.

## Data Availability

The data that support the findings of this study are available from the corresponding author upon reasonable request.

## Supplementary Materials

The following supporting information can be downloaded at: https://www.mdpi.com/article/10.3390/nu15143194/s1, Table S1: The results of AMSTAR-2 methodological quality assessment for each SR. Table S2: The essential characteristics of RCTs included in meta-analysis. Figure S1: Risk of bias summary. References [24,25,26,27,28,29,30,31,32,33,34,35,36,37,38,39,40,41,42,43,44,45,46] have been cited in Supplementary Materials.
