# Supplementary material for: The Effects of Selenium Supplementation in the Treatment of Autoimmune Thyroiditis: An Overview of Systematic Reviews
Source: Nutrients. 2023 Jul 19;15(14):3194. doi: 10.3390/nu15143194 (PMC10386011; doi:10.3390/nu15143194)
Supplement: Supplementary file 1 [file nutrients-15-03194-s001.zip › Supplementary Figure S1 Risk of bias summary.pdf]

	Random sequence generation (selection bias)	Allocation concealment (selection bias)	Blinding of participants and personnel (performance bias)	Blinding of outcome assessment (detection bias)	Incomplete outcome data (attrition bias)	Selective reporting (reporting bias)	Other bias
De Farias 2015 [24]	+	?	+	+	+	+	+
Deng 2013 [34]	+	?	?	+	+	+	+
Duntas 2003 [32]	?	?	?	+	+	+	+
Eskes 2014 [25]	+	?	+	+	+	+	+
Gartner 2002 [33]	−	?	+	+	+	+	?
Huang 2014 [43]	?	?	?	+	+	+	+
Kachouei 2018 [44]	?	?	+	+	+	+	+
Karanikas 2008 [29]	−	?	?	+	?	+	?
Karimi 2019 [45]	+	+	?	+	+	+	+
Krysiak 2011 A [27]	?	?	+	+	+	+	+
Krysiak 2011 B [27]	?	?	+	+	+	+	+
Krysiak 2012 A [26]	?	?	+	+	+	+	+
Krysiak 2012 B [26]	?	?	+	+	+	+	+
Liu 2019 [40]	+	?	?	+	+	+	+
Mazokopakis 2007 [30]	−	?	?	?	+	+	?
Nacamulli 2010 [28]	?	?	?	+	?	+	+
Pirola 2016 [42]	+	?	?	+	+	+	+
Shou 2013 [38]	−	?	?	+	+	+	?
Turker 2006 [31]	−	?	?	?	?	+	?
Wang 2018 A [46]	?	?	+	+	+	+	+
Wang 2018 B [46]	?	?	+	+	+	+	+
Wang 2020 [41]	−	?	?	?	+	+	?
Yan 2008 [37]	−	?	?	+	+	+	?
Zhang 2013 [36]	?	?	?	+	+	+	+
Zhou 2016 [39]	?	?	?	+	+	+	+
Zhu 2013 [35]	?	?	?	?	+	−	?
