# Supplementary material for: The Effects of Selenium Supplementation in the Treatment of Autoimmune Thyroiditis: An Overview of Systematic Reviews
Source: Nutrients. 2023 Jul 19;15(14):3194. doi: 10.3390/nu15143194 (PMC10386011; doi:10.3390/nu15143194)
Supplement: Supplementary file 1 [file nutrients-15-03194-s001.zip › Supplementary Table S2 The essential characteristics of RCTs included in meta-analysis.pdf]

Table S2. The essential characteristics of RCTs included in meta-analysis

study	Country	Sample size (int./con.)	Comparative groups	LT4	Age (years)		Female (%)	Duration (months)
					Int.	Con.		
De Farias 2015 [24]	Brazil	55 (28/27)	SeMet (200 μg/D) vs. placebo	Part	Median 48	Median 44	91	3
Eskes 2014 [25]	Netherlands	61 (30/31)	NaSe (200 μg/D) vs. placebo	None	Median 43.5 (20-68)	Median 45.0 (21-74)	100	6
Krysiak 2012 A [26]	Poland	73 (37/36)	SeMet (200 μg/D) vs. placebo	None	Mean 41 ± 3	Mean 40 ± 4	100	6
Krysiak 2012 B [26]	Poland	76 (38/38)	SeMet (200 μg/D) + LT4 vs. LT4	All	Mean 39 ± 4	Mean 42 ± 4	100	6
Krysiak 2011 A [27]	Poland	82 (42/40)	SeMet (200 μg/D) vs. placebo	None	Mean 40 ± 4	Mean 38 ± 3	100	6
Krysiak 2011 B [27]	Poland	83 (42/41)	SeMet (200 μg/D) + LT4 vs. LT4	All	Mean 39 ± 4	Mean 37 ± 3	100	6
Nacamulli 2010 [28]	Italy	76 (46/30)	NaSe (80 μg/D) vs. no treatment	None	Median 43 (17-75)	Median 43 (27-63)	86	12
Karanikas 2008 [29]	Austria	36 (18/18)	NaSe (200 μg/D) + LT4 vs. placebo + LT4	All	Mean 47 (19-85)		100	3
Mazokopakis 2007 [30]	Greece	80 (40/40)	SeMet (200 μg/D) vs. Se6 + no treatment6	Part	Median 37 (24-52)		100	12
Turker 2006 [31]	Turkey	88 (48/40)	SeMet (200 μg/day) + LT4 vs. placebo + LT4	All	Mean 40.8 ± 12.5	Mean 39.2 ± 14.4	100	3
Duntas 2003 [32]	Greece	65 (34/31)	SeMet (200 μg/D) + LT4 vs. placebo + LT4	All	Mean 47.8 (22-61)		86	6
Gartner 2002 [33]	Germany	70 (36/34)	NaSe (200 μg/day) + LT4 vs. placebo + LT4	All	Mean 41.6 ± 12.1	Mean 43.0 ± 12.1	100	3
Deng 2013 [34]	China	94 (48/46)	Se-yeast (200 μg/D) vs. placebo	None	Mean 39 ± 12	Mean 40 ± 12	86	6
Zhu 2013 [35]	China	96 (50/46)	Se-yeast (200 μg/D) + methimazole vs. methimazole	None	Mean 42.1 ± 13.6	Mean 43.4 ± 12.9	83	12
Zhang 2013 [36]	China	66 (46/20)	Se-yeast (200 μg/D) + LT4 vs. LT4	All	Mean 36.3 ± 11.1	Mean 39.3 ± 13.1	91	3

Yan 2008 [37]	China	114 (59/55)	NaSe (200 µg/day) + LT4 vs. LT4	All	Mean 43.8 ± 12.7	Mean 40.3 ± 11.2	88	3
Shou 2013 [38]	China	96 (46/50)	NaSe (200 µg/day) vs. no treatment	Part	Mean 40.3 ± 12.6		83	6
Zhou 2016 [39]	China	80 (40/40)	Se-yeast (200 µg/D) + methimazole vs. placebo + methimazole	None	Mean 36.25 ± 18.27		69	6
Liu 2019 [40]	China	102 (51/51)	Se-yeast (100 µg/D) + methimazole vs. methimazole	None	Mean 38.64 ± 6.71	Mean 37.86 ± 6.01	77	12
Wang 2020 [41]	China	105 (53/52)	Se-yeast (200 µg/D) vs. placebo	Part	Mean 36.36 ± 9.72	Mean 35.49 ± 9.07	57	6
Pirola 2016 [42]	Italy	192 (96/96)	SeMet (83 µg/D) vs. no treatment	None	Mean 32.2 ± 7.0	Mean 33.1 ± 6.4	63	4
Huang 2014 [43]	China	90 (45/45)	Se-yeast (200 µg/D) vs. placebo	None	Mean 34.8 ± 6.3	Mean 34.9 ± 6.1	68	6
Kachouei 2018 [44]	Iran	70 (35/35)	NaSe (200 µg/D) + LT4 vs. placebo + LT4	All	Mean 45.3 ± 10.8	Mean 45.6 ± 12.1	63	3
Karimi 2019 [45]	Iran	66 (38/28)	NaSe (200 µg/D) vs. placebo	Part	Mean 38.18 ± 13.29	Mean 39.32 ± 12.28	76	3
Wang 2018 A [46]	China	313 (153/160)	Se-yeast (200 µg/D) vs. placebo	None	Mean 41.4 ± 13.1		100	6
Wang 2018 B [46]	China	51 (28/23)	Se-yeast (200 µg/D) vs. placebo	Part	Mean 41.4 ± 13.1		100	6

Abbreviations: int./con., intervention group/control group; Semet, selenomethionine; NaSe, sodium selenite.
